# UNDERSTANDING THE CHARACTERISTICS AND WELL-BEING OF AMERICAN INDIAN AND ALASKA NATIVE GRANDPARENT CAREGIVERS

**DOI:** 10.1093/geroni/igac059.2053

**Published:** 2022-12-20

**Authors:** Ramona Danielson, Shelly Davis, Collette Adamsen

**Affiliations:** North Dakota State University, Fargo, North Dakota, United States; University of North Dakota School of Medicine & Health Sciences, Grand Forks, North Dakota, United States; University of North Dakota, Grand Forks, North Dakota, United States

## Abstract

**Background:**

American Indian/Alaska Native (AI/AN) grandparents have always had an important role in their grandchildren’s lives. Grandparents being the primary caregiver of their grandchildren has become a more pronounced occurrence in AI/AN populations in recent years and warrants review.

**Methods:**

Data come from the National Resource Center on Native American Aging’s 2017-2020 needs assessment of AI/AN adults ages 55+. Analysis explored demographic and well-being indicators by caregiving status (N=19,855): not a caregiver of a grandchild (non-CG; 71%); part-time caregiver (PT-CG; 18%); and primary caregiver (PR-CG; 11%).

**Results:**

Caregivers were more likely to be younger, be married, have higher education and incomes, and live with family in a single-family residence than non-CG. PR-CG were more likely to be female, employed full-time, and live with family on reservation/trust land than PT-CG and non-CG. Regarding indicators of well-being, caregivers were more likely to participate in cultural practices than non-CG. PT-CG were more likely to socialize and have recently participated in vigorous exercise, were less likely to have fair/poor health, and had fewer daily self-care restrictions than PR-CG and non-CG. PR-CG were more likely to be obese and daily smokers and had a higher measure of mental health concern, but were less likely to have recently binge drank or mainly eat alone than PT-CG and non-CG.

**Conclusions:**

The demographics reviewed were all significantly different. PT-CG had better outcomes, pointing to a potential protective benefit, while PR-CG had more mental health strain. Further research is needed to better understand the interconnectedness of the indicators analyzed.

